# Inclusion complexes of trihexyphenidyl with natural and modified cyclodextrins

**DOI:** 10.1186/s40064-015-0986-7

**Published:** 2015-05-07

**Authors:** Hideko Maeda, Risa Tanaka, Hirokazu Nakayama

**Affiliations:** Department of Functional Molecular Chemistry, Kobe Pharmaceutical University, 4-19-1 Motoyamakita-machi, Higashinada-ku, Kobe, 658-8558 Japan

**Keywords:** Trihexyphenidyl, Sulfobutylether-*β*-cyclodextrin, Inclusion complexes, Spectrofluorometry, Solubility

## Abstract

The solubility of trihexyphenidyl (Thp) was improved by its combination with *β*-cyclodextrin (*β−*CD) and modified *β*-CDs. The solubility of Thp was found to be increased in the presence of *β*-CD, hydroxypropyl-*β*-cyclodextrin (HP-*β*-CD), methyl-*β*-cyclodextrin (Me-*β*-CD) and sulfobutylether-*β*-cyclodextrin (SBE-*β*-CD). In particular, the solubility of Thp in conjunction with SBE-*β*-CD was increased by a factor of 11. The formation constant (*K*_*c*_) for the Thp/SBE-*β*-CD inclusion complex was determined to be 2300 L/mol based on fluorometry data. The structure of the Thp/SBE-*β*-CD complex in aqueous solution was examined by ^1^H-^1^H rotating frame nuclear Overhauser effect spectroscopy (ROESY) NMR, and the phenyl moiety of the Thp was found to coordinate with the secondary hydroxyl face of the SBE-*β*-CD. A solid Thp/SBE-*β*-CD inclusion complex was prepared by freeze-drying.

## Introduction

Trihexyphenidyl (Thp) is a pharmaceutical compound that has been shown to improve various disease symptoms, including muscle rigidity, finger tremors and depression, by regulating the release of adrenaline.

Natural cyclodextrins (*α*-, *β*- and *γ*-CD) are widely used in many fields since they readily form inclusion complexes with a variety of organic compounds (Saenger 1980; ÖZdemir and Ordu 1998; Reineccius et al. 2002). As an example, *β*-cyclodextrin (*β*-CD) is used to suppress the bitterness of antihistamine drugs in solution through the formation of inclusion complexes (Hibi et al. 1984; Ono et al. 2011). However, *β*-CD exhibits relatively low solubility in water, which limits its applications in pharmaceutical formulations. Therefore, various CD derivatives have been synthesized to extend the potential applications (Uekama 1985; Szejtli 1992; Loftsson and Duchêne 2007). We have previously reported complexes of *α*-lipoic acid and melatonin with modified CD derivatives (Maeda et al. 2010, 2013), and determined that the solubilities of *α*-lipoic acid and melatonin in the presence of sulfobutylether-*β*-CD increased by factors of 20 and 10, respectively.

In the present work, we investigated the solubility of inclusion complexes of Thp with natural and modified CDs at constant pH. To date, Thp complexes have not been assessed spectroscopically because of the low solubility of Thp. Haiyun *et al.* reported the evaluation of the complexation of rutin and *β*-CD by fluorescence spectroscopy in a phosphate solution containing 2% (v/v) methanol (Haiyun et al. 2003). Recently, Alvarez-Parrilla *et al.* reported stability constants for quercetin and rutin according to the methodology described by Haiyun (Alvarez-Parrilla et al. 2005). More recently, Al-Rawashdeh et al. reported the complexation of the sunscreen agents (oxybenzene, E-2-ethylhexyl-methoxycinnamate, octocrylene) and *β*-CD by UV-Vis spectroscopy in methanol/water mixture. The results demonstrate that the formation of inclusion complexes of the sunscreen agents and *β*-CD can inhibit the photodegradation (Al-Rawashdeh et al. 2010, 2013). Therefore, this method using an organic solvent was used in the present study to allow determination of the formation constants (*K*_c_) of Thp with various CDs.

The enantiodiscrimination of chiral Thp enantiomers by carboxylated methyls of *α*-, *β*-, and *γ*-CD was predicted by molecular docking study (Mulisa et al. 2014). More recently, structure of Thp/*β*-CD was elucidated by ^1^H NMR spectroscopic and computational methods (Ali and Shamim 2014).

The stability constant (*K*) and *K*_c_ values of Thp with various CDs were obtained in a 50 mmol pH 7 phosphate buffer containing ethanol. At present, there are no solubility data or spectroscopic data for such inclusion complexes at constant pH in the literature. Therefore, we first investigated the effect of the natural CDs and then examined a series of modified CDs: sulfobutylether-*β*-cyclodextrin (SBE-*β*-CD), hydroxypropyl-*α*-cyclodextrin (HP-*α*-CD), hydroxypropyl-*β*-cyclo dextrin (HP-*β*-CD), hydroxypropyl-*γ*-cyclodextrin (HP-*γ*-CD) and methyl-*β*- cyclodextrin (Me-*β*-CD) on the solubility of Thp.

## Materials and methods

### Materials

HP-*α*-CD, HP-*β*-CD, HP-*γ*-CD and Thp were purchased from Sigma-Aldrich (St. Louis, USA), *α*-CD, *β*-CD and *γ*-CD were purchased from Wako Chemical (Tokyo, Japan) and SBE-*β*-CD and Me-*β*-CD were purchased from Cydex (Kansas, USA) and the Junsei Chemical Co., Ltd. (Tokyo, Japan), respectively.

### Phase solubility study

CD solutions of varying concentrations were made in a 10:90 (v/v) ethanol/50 mmol phosphate buffer mixture and were combined with excess amounts of Thp, after which the solutions were stirred at 300 rpm and 25°C. The concentration of Thp in each solution was subsequently measured using a fluorescence spectrometer following 3, 17, 24, 48 and 72 h. The data showed that an equilibrium concentration was obtained after 72 h, and so at that point the solutions were filtered through a 0.45 *μ*m membrane. The concentration of Thp in each solution was determined based on fluorescence at λ = 287 nm with excitation at λ = 257 nm, using a Shimazu RF-5300PC spectrometer.

### Spectrometry

The pH of each sample solution was maintained at 7 by the addition of a 50 mmol/L potassium dihydrogen phosphate buffer. A 5.0 × 10^-3^ mol/L solution of Thp in ethanol was prepared and used in all experimental trials. In these trials, a 1 mL aliquot of this stock solution was transferred into a 10 mL volumetric flask together with an appropriate amount of a 1.0 × 10^-2^ mol/L and 1.25 × 10^-2^ mol/L CD phosphate solution, giving a final Thp concentration of 5.0 × 10^-4^ mol/L and CD concentrations of nil to 1.0 × 10^-2^ mol/L. Each solution was subsequently filtered through a 0.45 *μ*m membrane. The fluorescence spectra of Thp (5.0 × 10^-4^ mol/L) was measured in a 10:90 (v/v) ethanol/50 mmol phosphate buffer solution. The fluorescence intensity generated by the Thp was found to vary depending on the concentration of CD added. Therefore, the value of *K*_*c*_ could be obtained from the differences in the fluorescence intensities of the Thp/CD solutions. Fluorescence spectra were recorded with a Shimazu RF-5300 PC spectrometer.

### Sample preparation

Solid complexes of Thp and CDs were prepared by the following methods. Simple mixtures of Thp and CDs in solid form were prepared for comparison purposes.

#### Physical mixing method

Solid CD and Thp were combined at a ratio of 1 mmol (2.16 g in the case of the SBE-*β*-CD) to 1 mmol (0.3379 g for Thp) within a nylon bag, after which the mixture was shaken for 30 min.

#### Kneading method

Solid CD and Thp were combined at a ratio of 1 mmol (2.16 g for SBE-*β*-CD) to 1 mmol (0.3379 g for Thp) together with a small amount of ethanol (ca. 3 mL) and kneaded for 30 min.

#### Freeze-drying method

Solid CD and Thp were combined at a ratio of 10 mmol (1.08 g for SBE-*β*-CD) to 10 mmol (0.168965 g for Thp) and dissolved in 50 mL of phosphate buffer (pH 7), following which the water was removed under vacuum at -40°C.

### Analytical methods

#### ^1^H NMR measurements

A mixture of CD and Thp at a ratio of 10 mmol (0.0216 g for SBE-*β*-CD) to 10 mmol (0.0034 g for Thp) was dissolved in 1 mL of D_2_O to allow for ^1^H- and ^1^H-^1^H ROESY NMR measurements. When analyzing solely Thp, the compound was instead dissolved in CD_3_OD because of its poor solubility in water, using a ratio of 10 mmol of Thp to 1 mL of CD_3_OD.

^1^H-^1^H ROESY NMR data were obtained in the phase sensitive mode under continuous wave (CW) operation, with spin lock for mixing. Spectra of the inclusion complex were obtained by spin lock pulses of 180_x_-180_-x_ with a steady-state sequence prior to d1, together with [grad]_z_-90_x_-[grad]_z_ pulses.

#### X-ray diffraction (XRD)

Powder XRD patterns were obtained using a Rigaku Denki (Tokyo, Japan) Rint 2000 diffractometer with Ni-filtered Cu K*α* radiation.

#### Differential scanning calorimetry (DSC)

The thermal behaviors of the solid complexes were assessed using a DSC 3100SA instrument (NETZSCH, Selb, Germany) at a heating rate of 10°C/min from 25 to 500°C.

## Results and discussion

### Phase solubility

**Figure 1:**
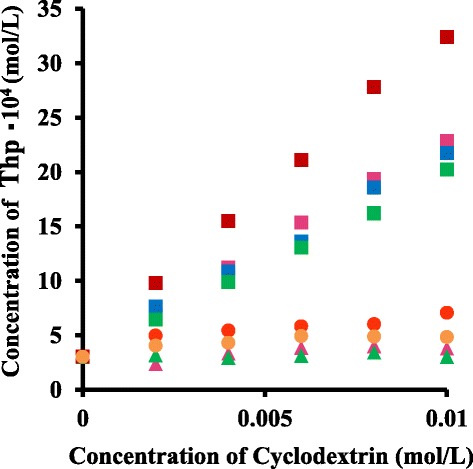
Phase solubility diagrams at 25°C and pH 7. The solubility of Thp in the presence of SBE-*β*-CD (Red square), Me-*β*-CD (Blue square), HP-*β*-CD (Green square), *β*-CD (Pink square), HP-*γ*-CD (Yellow circle), HP-*α*-CD (Green triangle), *α*-CD (Pink triangle) and *γ*-CD (Red circle) are shown as a function of their concentration.

The complexation of Thp in the presence of natural CDs and various modified CDs was examined using the solubility method (Higuchi and Connors 1965). Figure 1 shows the phase solubility diagram of various Thp/CD complexes at 25°C and pH 7. A considerable increase in the solubility of Thp was observed in the presence of *β*-CD and the *β*-CD derivatives. In particular, in the presence of SBE-*β*-CD, the solubility of Thp was 11 times higher than that of Thp itself. Based on the Higuchi-Connors theory (Higuchi and Connors 1965), both *β*-CD and the *β*-CD derivatives showed an A_L_-type solubility curve, indicating the formation of soluble complexes. There was no improvement in the solubility when employing either *α*-CD or HP-*α*-CD, while the use of *γ*-CD and HP-*γ*-CD approximately doubled the solubility of the Thp.

Table 1 summarizes the *K* values calculated from regression analysis of the phase solubility curves, using Eq. (1). Note that *K* values were not obtained for the *γ*-CD and HP-*γ*-CD solutions since these solutions did not generate linear solubility curves.

**Table 1:** **Regression parameters and stability constants (** ***K*** **) for Thp/CD complexes as determined by solubility diagrams at pH 7**

**CD**	**Slope**	**Intercept**	**R** ^**2**^	***K*** **(L/mol)**
*β*-CD	0.199	3.14	0.999	800 ± 50
HP-*β*-CD	0.169	3.00	0.999	680 ± 40
Me-*β*-CD	0.185	3.33	0.994	680 ± 80
SBE-*β*-CD	0.295	3.51	0.998	1200 ± 200

1 $$ K\kern0.5em =\kern0.5em \frac{\mathrm{slope}}{\operatorname{int}\mathrm{ercept}\kern0.5em \left(1\kern0.5em \hbox{-} \kern0.5em \mathrm{slope}\right)} $$

The complexation ability of the SBE-*β*-CD appears to be the highest among the CDs investigated in this study.

### Spectrophotometry

**Figure 2:**
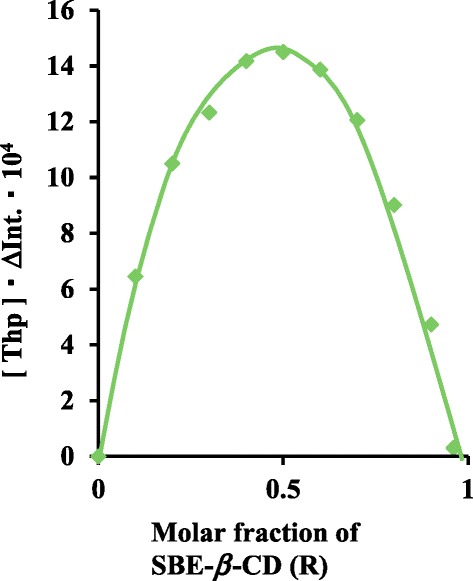
Job’s plot of Thp/SBE-*β*-CD from fluorescence intensity measurements in a 50:50 (v/v) ethanol/50 mmol phosphate buffer solution.

Solutions containing 1.0 × 10^-4^ mol/L of Thp and 1.0 × 10^-4^ mol/L SBE-*β*-CD were made in a 50:50 (v/v) ethanol/50 mmol phosphate buffer solution and samples for the preparation of a Job’s plot were prepared by mixing these solutions in varying proportions. The resulting Job’s plot (Job 1928) obtained from fluorescence intensity measurements of these Thp/SBE-*β*-CD solutions is presented in Figure 2. The maximum [Thp] · *Δ*Intensity value at *R* = 0.5 indicates the formation of a complex in which the stoichiometry is 1:1. The stoichiometries of the other Thp/CD complexes were also found to be 1:1 using this same method.

**Figure 3:**
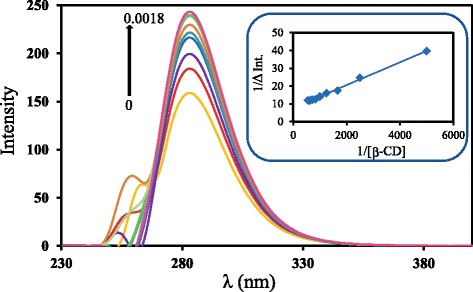
Fluorescence spectra of Thp in the presence of *β*-CD. The concentration of Thp is 5.0 × 10^-4^ mol/L and concentrations of *β*-CD are 0, 0.2, 0.4, 0.6, 0.8, 1.0, 1.2, 1.4, 1.6 and 1.8 × 10^-3^ mol/L from bottom to top in 10:90 (v/v) ethanol/50 mmol phosphate buffer solution. Inset shows their Benesi-Hildebrand plot for fluorescence increments at 283 nm.

Figure 3 shows the fluorescence spectra obtained for Thp in combination with various *β*-CD concentrations, in which the fluorescence intensity at 283 nm is seen to increase with increasing *β*-CD concentrations from nil to 1.8 × 10^-3^ mol/L. From these data, the *K*_*c*_ value of the complex of Thp with each CD could be determined from a plot of the reciprocal of *ΔF* against the reciprocal of [CD] according to Benesi’s equation (Benesi and Hildebrand 1949), below.

2 $$ \frac{1}{\varDelta F}=\frac{1}{\mathrm{a}\left[\mathrm{G}\right]\bullet {K}_c}\bullet \frac{1}{\left[\mathrm{C}\mathrm{D}\right]}+\frac{1}{\mathrm{a}\left[\mathrm{G}\right]} $$

Here *ΔF* is calculated as:

3 $$ \varDelta F=F\hbox{--} {F}_0 $$

where *F* and *F*_*0*_ are the fluorescence intensities in the presence and absence of the CD, respectively, while [G], [CD] and a are the concentration of the guest compound (Thp), the concentration of the CD and a proportionality constant (Kondo et al. 1976; Hamai 1982), respectively.

The inset in Figure 3 demonstrates that a plot of 1/*ΔF* at 283 nm as a function of 1/[CD] for the *β*-CD data generates a straight line that in turn gives a *K*_*c*_ value of 1200 L/mol.

**Figure 4:**
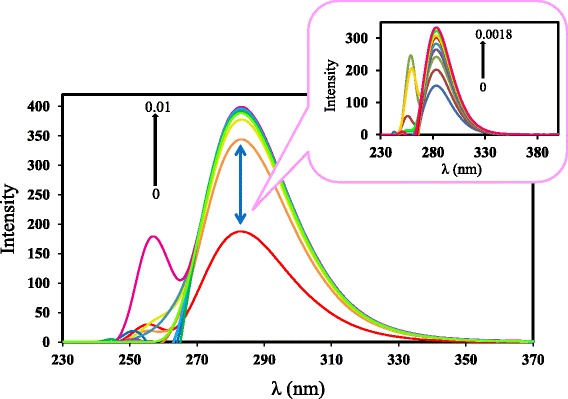
Fluorescence spectra of Thp in the presence of SBE-*β*-CD. The concentration of Thp is 5.0 × 10^-4^ mol/L and concentrations of SBE-*β*-CD are 0, 0.1, 0.2, 0.3, 0.4, 0.5, 0.6, 0.7, 0.8, 0.9, 1.0 × 10^-2^ mol/L from bottom to top in 10:90 (v/v) ethanol/50 mmol phosphate buffer solution. Inset shows fluorescence spectra in the presence of SBE-*β*-CD concentrations from bottom to top are 0, 0.2, 0.4, 0.6, 0.8, 1.0, 1.2, 1.4, 1.6 and 1.8 × 10^-3^ mol/L.

Figure 4 presents the fluorescence spectra obtained for Thp together with various SBE-*β*-CD concentrations ranging from nil to 1.0 × 10^-2^ mol/L, while the associated inset shows the data for SBE-*β*-CD concentrations from nil to 1.8 × 10^-3^ mol/L. A plot of 1/*ΔF* at 283 nm against [CD] for these data again gives a straight line, from which a *K*_*c*_ value of 2300 L/mol was obtained.

Table 2 summarizes the *K*_*c*_ values estimated from Eq. (2). Based on the data in Table 1, obtained using the solubility method, the original *β*-CD and the *β*-CD derivatives were all found to improve the solubility of the Thp. From the spectral data in Table 2, the complexation ability of the SBE-*β*-CD appears to be the highest among the CDs used in this study. Compared with our stability constants (*K*) obtained by the solubility method, the *K*_*c*_ values of Thp/*β*-CD and Thp/SBE-*β*-CD was somewhat large, while the *K*_*c*_ values of the Thp/Me-*β*-CD and Thp/HP-*β*-CD complexes are seen to be similar to the *K* values obtained by the solubility method. Apparent stability constant estimated from solubility data has large error and has been used rough estimate of complex formation. Therefore, the difference between the stability constants obtained from solubility diagrams and spectroscopic has been sometimes recognized.

**Table 2:** **Regression parameters and formation constants (** ***K***_***c***_ **) for Thp/CD complexes as determined from spectra acquired in a 10% (v/v) ethanol phosphate solution**

**CD**	**Slope**	**Intercept**	**R** ^**2**^	***K*** **(L/mol)**
*β*-CD*	6.4 × 10^-3^	7.74	0.996	1200 ± 40
HP-*β*-CD**	5.4 × 10^-3^	3.78	0.992	700 ± 20
Me-*β*-CD**	5.0 × 10^-3^	3.87	0.972	770 ± 40
SBE-*β*-CD**	1.9 × 10^-3^	4.41	0.967	2300 ± 200

*[CD] = 0 – 0.002 mol/L, **[CD] = 0 – 0.01 mol/L.

The solubility of Thp in the presence of SBE-*β*-CD was increased by a factor of 11, and the stability of the resulting Thp/SBE-*β*-CD complex was higher than those of the other Thp/CD complexes. Because SBE-*β*-CD has sulfobutylether chains on both sides of CD rings, it has large hydrophobic space than the other derivatives of CDs, it would solubilize Thp. Furthermore, the end of sulfobutylether chain is anionic ion, which also increase the solubility of Thp/SBE-*β*-CD complex. Therefore, the structure of Thp/SBE-*β*-CD complex was investigated.

### Structure of the inclusion complex

**Figure 5:**
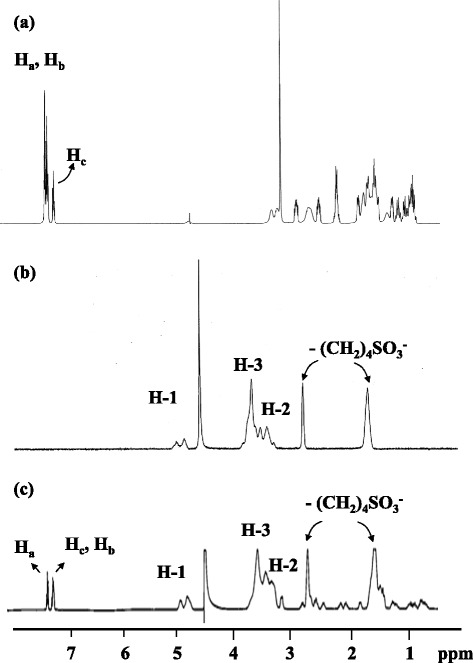
^1^H NMR spectra of **(a)** Thp **(b)** SBE-*β*-CD **(c)** Thp /SBE-*β*-CD complex. Note: the signal at 3.3 ppm **(a)** is due to residual methanol.

Figures 5(a to c) present the ^1^H NMR spectra of Thp, SBE-*β*-CD and the Thp/SBE-*β*-CD complex respectively, acquired in CD_3_OD (Thp) and D_2_O (SBE-*β*-CD and Thp/SBE-*β*-CD). The presence of peaks attributable to Thp in Figure 5(c) demonstrates the successful formation of the complex.

**Figure 6:**
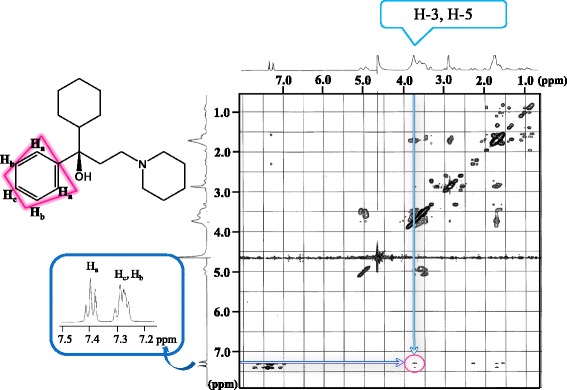
^1^H-^1^H ROESY spectrum of the Thp/SBE-*β*-CD complex.

Figure 6 shows the ^1^H-^1^H ROESY NMR spectrum of the Thp/SBE-*β*-CD inclusion complex. This spectrum exhibits correlations between the signals at 7.39, 7.28 and 7.29 ppm (assigned to H_a_, H_b_ and H_c_ of Thp based on ^1^H-^1^H COSY NMR) and those at 3.7 ppm (H-3 and H-5 of the SBE*-β*-CD). These results indicate that the Thp phenyl group was incorporated into the cavity of the SBE-*β*-CD complex, whereas the Thp cyclohexane ring and piperidine ring was not incorporated. From these data, the stoichiometry of the Thp/SBE-*β*-CD complex was determined to be 1:1.

The above NMR data indicate that the Thp/SBE-*β*-CD complex consisted of an inclusion structure in the solution state. To further investigate this complex, a Thp/SBE-*β*-CD solid complex was subsequently synthesized and characterized.

### Characterization of the Thp/SBE-*β*-CD solid complex

**Figure 7:**
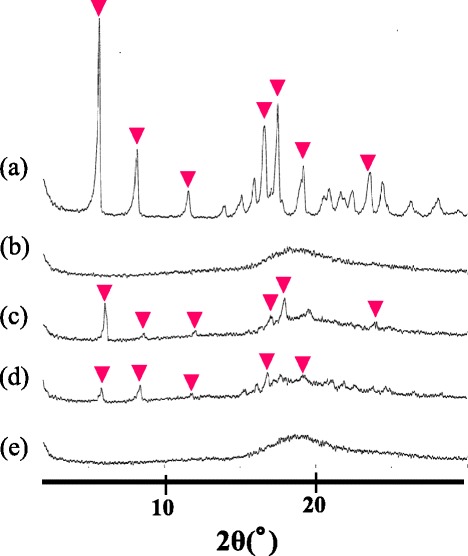
XRD patterns of **(a)** Thp, **(b)** SBE-*β*-CD and the Thp/SBE-*β*-CD (1:1) solid systems obtained by **(c)** physical mixing, **(d)** kneading and **(e)** freeze-drying. Red-down pointing triangle shows the peaks due to crystalline Thp.

Figure 7 presents the XRD patterns of Thp, SBE-*β*-CD and the Thp/SBE-*β*-CD (1:1) solid systems obtained by physical mixing, kneading and freeze-drying methods. In Figure 7(a), seven characteristic diffraction peaks resulting from crystalline Thp are present at 2*θ* values of 5.69, 8.21, 11.6, 16.6, 17.5, 19.2 and 23.6°. In contrast, the XRD pattern of SBE-*β*-CD (Figure 7(b)) shows a broad diffraction profile because the solid material is amorphous due to the random substitution pattern of the sulfobutylether groups.

The solid systems obtained by physical mixing and kneading (Figures 7(c and d)) show six peaks at 2*θ* values of 6.07, 8.64, 12.1, 17.1, 17.9 and 24.0°, and five peaks at 2*θ* values of 5.85, 8.40, 11.9, 16.8 and 19.1° due to crystalline Thp, respectively. The solid obtained by the freeze-drying method, however, (Figure 7(e)) exhibits an amorphous state. These data suggest that the solid complex was obtained via freeze-drying and that Thp was included in the SBE-*β*-CD cavities.

**Figure 8:**
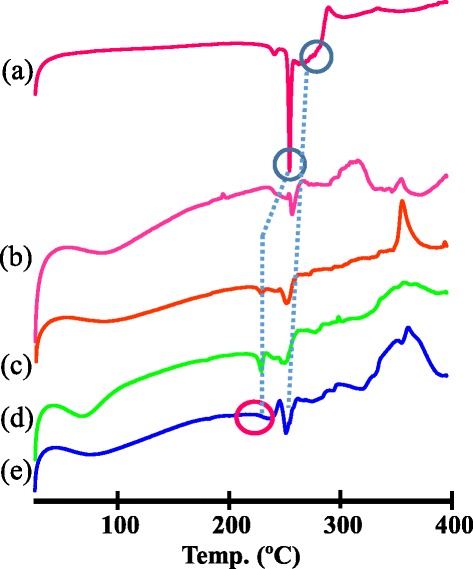
DSC curves of **(a)** Thp, **(b)** SBE-*β*-CD and Thp/SBE-*β*-CD (1:1) solid systems obtained by **(c)** physical mixing, **(d)** kneading and **(e)** freeze-drying.

Figure 8 shows the DSC curves obtained for Thp, SBE-*β*-CD and the Thp/SBE-*β*-CD (1:1) solid complexes obtained by the physical mixing, kneading and freeze-drying methods. An endothermic peak at approximately 256°C, corresponding to the melting point of Thp, is observed in the Thp curve (Figure 8(a)), as well as for the solid systems obtained by physical mixing (Figure 8(c)) and kneading (Figure 8(d)). The melting points of the physically mixed and kneaded materials were 229.5 and 231.5°C, respectively. An endothermic peak representing the decomposition of SBE-*β*-CD can be seen at approximately 260°C in Figure 8(b), as well as in the curves obtained from the physically mixing, kneaded and freeze-dried samples (Figures 8(c to e)). The decomposition points of the solid systems obtained by the physical mixing, kneading and freeze-drying methods were 253.7, 256.9 and 253.9°C, respectively.

In the case of the freeze-dried solid complex, the endothermic peak corresponding to the melting of Thp is not present, suggesting that the Thp interacts with the SBE-*β*-CD in the solid state to form an inclusion complex.

These results demonstrate that the solid complex obtained by the freeze-drying method was completely different from the systems generated using physical mixing and kneading. The Thp evidently interacts with SBE-*β*-CD in the solid complex obtained by the freeze-drying method, forming an inclusion complex.

## Conclusion

The effects of natural and various modified CDs on the solubility of Thp were assessed, using the solubility method. The solubility of Thp in the presence of SBE-*β*-CD was found to be increased significantly, by a factor of approximately 11. The stoichiometry of each Thp/CD complex was observed to be 1:1. In the case of the Thp/SBE-*β*-CD inclusion complex, the formation constants (*K*_*c*_) obtained by fluorometry was 2300 L/mol. The phenyl groups of the Thp were found to be included in the SBE-*β*-CD cavities. Finally, the freeze-drying method was determined to successfully generate solid inclusion complexes.
